# The Association between Health Behaviours and Academic Performance in Canadian Elementary School Students: A Cross-Sectional Study

**DOI:** 10.3390/ijerph121114857

**Published:** 2015-11-20

**Authors:** Jessie-Lee D. McIsaac, Sara F. L. Kirk, Stefan Kuhle

**Affiliations:** 1 Applied Research Collaborations for Health, School of Health and Human Performance, Dalhousie University, 1318 Robie Street, Halifax, NS B3H 4R2, Canada; E-Mails: jessie-lee.mcisaac@dal.ca (J.-L.D.M.); sara.kirk@dal.ca (S.F.L.K.); 2 Perinatal Epidemiology Research Unit, Departments of Pediatrics and Obstetrics & Gynaecology, Dalhousie University, 5980 University Avenue, Halifax, NS B3K 6R8, Canada

**Keywords:** child, academic performance, diet, physical activity, Canada

## Abstract

*Background*: Establishing early healthy eating and physical activity behaviours is critical in supporting children’s long-term health and well-being. The objective of the current paper was to examine the association between health behaviours and academic performance in elementary school students in a school board in Nova Scotia, Canada. *Methods*: Our population-based study included students in grades 4–6 across 18 schools in a rural school board. Diet and physical activity were assessed through validated instruments. Academic performance measures were obtained from the school board for Mathematics and English Language Arts (ELA). Associations between health behaviours and academic performance were assessed using multilevel logistic regression. *Results*: Students with unhealthy lifestyle behaviours were more likely to have poor academic performance for both ELA and Mathematics compared to students with healthy lifestyle behaviours; associations were statistically significant for diet quality, physical activity, sugar-sweetened beverage consumption for ELA; and breakfast skipping, not being physically active at morning recess, and not being physically active after school for Mathematics. The effects of diet and physical activity were independent of each other and there was no interaction between the two exposures. *Conclusions*: Our findings suggest that support for healthy behaviours may help to improve academic outcomes of students.

## 1. Background

Establishing early healthy eating (HE) and physical activity (PA) behaviours is critical in supporting children’s long-term health and well-being. Globally, schools are acknowledged as an essential setting to develop and implement policies to support healthier lifestyle behaviours among children [1]. The connections between health and education are well established but the mechanisms that contribute to this relationship remain poorly understood [2]. Considering the increasing demands on schools to improve the academic performance of students while also supporting their health, it is important to better understand how an emphasis on HE and PA can help to support these academic priorities of student achievement [3,4]. Further research on the associations of health behaviours and academic performance will also help to clarify how current population health interventions in schools (e.g., HE and PA policies and programs) influence learning outcomes, so as to justify continued and future investment [5].

Eating a nutritious diet has been known to have a variety of benefits to the health of children and youth, especially on the development and health of brain structure and function [6]. A child’s nutritional status may be influenced by a diet that is lacking in overall food (nutrient insufficiency) or in important micronutrients (nutrient deficiency) that help the brain and body function [7], thereby having an impact on school performance. For example, food insufficiency has been associated with significantly lower standardized mathematics scores compared to students who reported having enough to eat [8].

Research has theorized that universal healthy breakfast programs and implementing nutrition policies can help to support the nutritional status of children by reducing nutritional deficiency or food insufficiency, which may help to improve school performance [7,9,10]. There is also evidence to support a relationship between energy expenditure through participation in PA on improved cognitive health and function [6]. Generally, acute physical exercise exerts a positive effect on cognition with linkages to school performance [11,12,13,14], as well as having an impact on attitudes and academic behaviour, including improved concentration, attention and classroom behaviour [12]. Studies have shown a long-term positive impact of moderate-to-vigorous PA [15] and that there may be an optimal amount of PA with too much time potentially resulting in poorer academic achievement [16,17,18,19]. Research measuring physical fitness has also demonstrated a linkage with school performance [11,20,21] with aerobic exercises appearing to yield the largest impact on children’s cognitive outcomes [11,21]. School-based PA strategies can help to support increased PA levels (e.g., physical education, extra-curricular activities, interscholastic sports and recess) have also demonstrated positive cognitive outcomes [11,12].

Although there is a plethora of literature describing associations between HE, PA and academic performance, the relationships need to be better characterized due to their synergistic nature [6]. Reviews of the literature have demonstrated different effects across studies, which might be a result of different measures or measurement of behaviours [9,11,22]. For example, there is robust evidence on the importance of breakfast consumption [9] compared to limited data on the influence of overall diet quality, fruit and vegetable consumption or other HE indices [7]. For PA, there is evidence to suggest that the volume or intensity of activity may be an important consideration [16], which should be considered separate from potential effects from aerobic fitness [11,22].

There is very little research that has analyzed the combined influence of health behaviours on academic performance. Martínez-Gómez *et al.* (2012) examined the independent and combined influence of meeting recommendations for four health behaviours among Spanish adolescents (PA, TV viewing, sleep and fruit intake) and found that girls with 3–4 healthy behaviours showed significantly higher odds of receiving passing grades for literature/language and mathematics compared with those with 0–1 health behaviours [23]. A population-level study of adolescents in Iceland examined the influence of health behaviours, body mass index (BMI), self-esteem and academic performance and the relationships. This research found positive associations between lower BMI, PA and good dietary habits with academic performance [24] and that BMI, diet and PA explained up to 24% of the variance in academic achievement [25]. Another study in the United States reported positive but different associations between higher standardized academic scores in mathematics and reading with various nutrition behaviours, PA and fitness [26]. Regression analysis also indicated that 11.1% and 6.7% of the variation in the mean mathematics and reading scores (respectively) could be accounted for by certain HE and PA behaviours, fitness and gender [26].

Considering the mixed results previously reported and the limited studies that have explored the relationship of both HE and PA behaviours, the objective of this study was to examine the individual and combined associations of health behaviours with academic performance in elementary school students in a school board in Nova Scotia, Canada.

## 2. Methods

The current analysis uses data from a population-based survey of students in grades 4–6 (about 9–12 years old) and their parents in a rural school board in Nova Scotia, Canada. Data collection was performed in spring 2014 and included information on students’ diet, PA and well-being and school culture through surveys with students, parents, school leaders and teachers, and an audit of the school environment. All schools with grade 4–6 students in this school board were invited to participate. Packages containing consent forms and a survey that were sent home with all students to obtain parental consent. All 18 eligible schools agreed to participate and parental consent was obtained for 670 students resulting in an average response rate of 46%.

### 2.1. Data Collection

Trained research assistants visited schools to administer a slightly modified, Canadian version of the Harvard Youth Adolescent Food Frequency Questionnaire (YAQ) to participating students [27], along with an additional survey that assessed PA and sedentary behaviour, self-efficacy, and quality of life. All participating students completed the questionnaires by themselves in their classroom as a group. Students were instructed not to discuss the survey with their classmates. Research assistants read the survey aloud, provided assistance when needed and responded to questions to support comprehension. The research assistants measured the heights and weights of participating students. Standing height was measured to the nearest 0.1 cm after students had removed their shoes. Body weight was measured in light indoor clothing to the nearest 0.1 kg on calibrated digital scales. The parent survey contained questions on sociodemographic factors, the home environment, their child’s health and their HE and PA behaviours.

### 2.2. Exposures

The main exposures of interest were the Diet Quality Index—International (DQI) score and the summary score of the Physical Activity Questionnaire for Children (PAQ-C). The DQI is a composite score ranging from 0 to 100 that includes aspects of diet adequacy, variety, balance, and moderation with higher scores indicating better diet quality [28]. This score was calculated based on student responses on their intake of 100+ food items in the YAQ. The estimated intake of each item was linked with information from the Canadian Nutrient File [29] to determine food group and nutrient intakes for the calculation of the DQI. The YAQ was developed for children aged 9 to 18 and gathers information on usual dietary intake and habits pertaining to mealtime behaviour [27]. The tool has been tested to demonstrate validity and reproducibility [30]. The PAQ-C was used to assess PA. The tool is a self-administered, 7-day recall instrument that was validated for children ages 8–14 and developed to assess general levels of PA throughout the school year for elementary students, including questions that assessed PA time during and after school and on the weekends [31]. The tool was validated in Canadian children and adolescents and has demonstrated acceptable to good reliability and validity for item and scale properties [31,32,33,34]. A PA score obtained from the PAQ-C ranges from 1 (low) to 5 (high) and was calculated from the mean score of nine questions related to frequency and intensity of PA at different times throughout the day. The DQI and PAQ-C scores were grouped into tertiles for the analysis. We also created a 4-level diet quality/PA variable to examine the individual and combined effects of the two main exposures (1 = DQI and PAQ-C high or medium tertile, 2 = PAQ-C low tertile and DQI high or medium tertile, 3 = DQI low tertile and PAQ-C high or medium tertile, 4 = both DQI and PAQ-C low tertile).

Secondary exposures included student-reported diet quality as measured by the Youth Healthy Eating Index (YHEI) [35] (in tertiles); fruit and vegetable and milk/dairy food group intake (meeting Canada’s Food Guide [36] recommendations yes/no); sugar-sweetened beverage consumption (<1 *vs.* ≥1 per day); and skipping breakfast (yes/no). We also included the parent reported exposures of being physically active with and without an instructor, respectively (≥4× *vs.* <4× per week); being physically active at morning recess and after school, respectively (≥4× *vs.* <4× per week); and screen time (≤2 h *vs.* >2 h per day).

### 2.3. Outcomes

The primary outcome was academic performance for Mathematics and English Language Arts (ELA) in the 2013/14 school year. Grades were obtained directly from the school board for each of the three terms. Grades (ranging from “A” to “D”) were transformed to a numeric scale (1 to 4), and the median of the three terms was used as the overall grade estimate for the academic year. Grades were then dichotomized with grades “C” (3) or “D” (4) being considered poor academic performance.

### 2.4. Covariates

Other variables used in the analysis were gender, household income (4 levels: $0 to $40,000; $40,001 to $60,000; $60,001 to $100,000; >$100,000 CDN; 21% had either missing values or parents had indicated that they preferred not to answer), parental education attainment (3 levels: Secondary school or less; College; University) and area of residence (urban *vs.* rural; based on the second character of the Forward Sortation Area in the Canadian postal code; rural postal codes contain a 0 as the second character). BMI was categorized as not overweight and not obese; overweight; and obese based on the cutoffs of the International Obesity Task Force [37].

### 2.5. Statistical Analysis

Sample characteristics were summarized by Mathematics and ELA academic performance. A series of multiple logistic regression models with school as the random effect was used to examine the associations between health behaviours and academic performance. Regression models were adjusted for household education and income, models with dietary exposures were further adjusted for energy intake [38]. Children with energy intakes <500 kcal or >5000 kcal were excluded from analyses of dietary exposures.

As response rates varied by area-level household income, response weights were calculated to reduce potential non-response bias. Using postal code-area population counts and average household incomes from the 2011 Census of Canada, we calculated response rates per decile of area-level household income and converted them into response weights. All analyses were weighted to the population of the Tri-County Regional School Board.

The statistical analysis was performed using Stata/SE 13 (Stata Corp., College Station, TX, USA).

### 2.6. Ethics and Consent

Ethics approval for this study was obtained from the Health Sciences Research Ethics Board at Dalhousie University (file # 2013-3094). Informed written consent was obtained from the parents of participating children; children provided written assent. Permission for data collection was also granted from the participating school board.

## 3. Results

The parents of 590 of the 670 students consented to linking the child's academic performance information with the study data. Of these, 535 had complete information on the primary exposures DQI and PAQ-C; their characteristics are summarized in Table 1.

Results from the multiple regression analysis for academic performance in ELA (Table 2) and Mathematics (Table 3) show that unhealthy lifestyle behaviours are positively associated with poor academic performance in both ELA and Mathematics; the associations were statistically significant in the adjusted models for DQI (low *vs.* high tertile), YHEI (low *vs.* high tertile), consumption of ≥1 sugar-sweetened beverage, PAQ-C score (low *vs.* high tertile) for ELA; and breakfast skipping, not being physically active at morning recess, and not being physically active after school for Mathematics. When DQI, PAQ-C, and screentime were entered into the model for ELA simultaneously, the odds ratio for screentime >2 h increased from 0.77 to 1.69 compared to the individual models but was not statistically significant. Conversely, in the combined model for mathematics, the odds ratio for PAQ-C screentime >2 h decreased from 1.45 to 0.74. The effect estimates for DQI and PAQ-C did not change substantially in either model. There was no additive interaction between diet quality and PA for either ELA or mathematics as the combined effect of having both low diet quality and low PA did not exceed the sum of the individual effects (data not shown).

**Table 1:** Sociodemographic characteristics and weight status of the 535 children in the study.

Characteristic	Prevalence
**Male Sex**	47%
**Grade**	
4	33%
5	38%
6	29%
**Rural Residence**	63%
**Household Income**	
≤$40,000	24%
$40,001–$60,000	15%
$60,001–$100,000	25%
>$100,000	14%
Missing	22%
**Household Education**	
Secondary school or less	25%
College	49%
University	26%
**Weight Status**	
Not overweight not obese	56%
Overweight	27%
Obese	17%

**Table 2:** Odds ratios (OR) and 95% confidence intervals (CI) for the association between health behaviours and poor academic performance in English Language Arts. Models were adjusted for household education and income, models with dietary exposures were further adjusted for energy intake.

	Univariate	Adjusted
	OR (95% CI)	OR (95% CI)
**Individual Associations**		
**Diet Quality Index**		
Low tertile	**5.16 (1.92–13.83)**	**4.26 (1.28–14.22)**
Medium tertile	1.70 (0.62–4.69)	1.18 (0.37–3.73)
High tertile	1.00 (Ref)	1.00 (Ref)
**Youth Healthy Eating Index**		
Low tertile	**4.03 (1.56–10.42)**	**3.22 (1.02–10.12)**
Medium tertile	**2.19 (1.07–4.47)**	1.48 (0.62–3.56)
High tertile	1.00 (Ref)	1.00 (Ref)
**Meets Fruit & Vegetable Intake Recommendations**		
No	1.83 (0.73–4.57)	1.46 (0.49–4.36)
Yes	1.00 (Ref)	1.00 (Ref)
**Meets Dairy Intake Recommendations**		
No	0.97 (0.46–2.04)	0.73 (0.31–1.71)
Yes	1.00 (Ref)	1.00 (Ref)
**≥1 Sugar-Sweetened Beverage per Day**		
Yes	**3.18 (1.51–6.71)**	**2.42 (1.10–5.34)**
No	1.00 (Ref)	1.00 (Ref)
**Skips Breakfast**		
Yes	0.72 (0.28–1.89)	0.87 (0.29–2.60)
No	1.00 (Ref)	1.00 (Ref)
**Physical Activity Questionnaire for Children**		
Low tertile	**2.49 (1.41–4.39)**	**2.10 (1.02–4.34)**
Medium tertile	1.08 (0.62–1.88)	0.92 (0.47–1.81)
High tertile	1.00 (Ref)	1.00 (Ref)
**Physically Active without Instructor <4×/Week**		
Yes	**3.59 (1.05–12.21)**	3.10 (0.73–13.20)
No	1.00 (Ref)	1.00 (Ref)
**Physically Active with Instructor <4×/Week**		
Yes	1.22 (0.73–2.04)	1.25 (0.75–2.10)
No	1.00 (Ref)	1.00 (Ref)
**>2 h of Screentime**		
Yes	1.08 (0.53–2.21)	0.77 (0.40–1.50)
No	1.00 (Ref)	1.00 (Ref)
**Physically Active at Morning Recess**		
No	1.34 (0.74–2.44)	1.08 (0.55–2.13)
Yes	1.00 (Ref)	1.00 (Ref)
**Physically Active after School**		
No	1.46 (0.69–3.09)	1.43 (0.61–3.34)
Yes	1.00 (Ref)	1.00 (Ref)
**Combined Associations**		
**Diet Quality Index**		
Low tertile	**4.79 (1.61–14.24)**	**4.41 (1.19–16.36)**
Medium tertile	1.65 (0.55–4.93)	1.18 (0.37–3.82)
High tertile	1.00 (Ref)	1.00 (Ref)
**Physical Activity Questionnaire for Children**		
Low tertile	**2.54 (1.37–4.72)**	**2.15 (1.14–4.06)**
Medium tertile	1.06 (0.52–2.13)	0.89 (0.44–1.82)
High tertile	1.00 (Ref)	1.00 (Ref)
**>2 h of Screentime**		
Yes	1.24 (0.63–2.41)	1.69 (0.87–3.28)
No	1.00 (Ref)	1.00 (Ref)

**Table 3:** Odds ratios (OR) and 95% confidence intervals (CI) for the association between health behaviours and poor academic performance in Mathematics. Models were adjusted for household education and income, models with dietary exposures were further adjusted for energy intake.

	Univariate	Adjusted
	OR (95% CI)	OR (95% CI)
**Individual Associations**		
**Diet Quality Index**		
Low tertile	2.07 (0.61–7.02)	1.68 (0.52–5.45)
Medium tertile	1.24 (0.36–4.31)	1.00 (0.30–3.33)
High tertile	1.00 (Ref)	1.00 (Ref)
**Youth Healthy Eating Index**		
Low tertile	2.27 (0.93–5.57)	1.71 (0.62–4.67)
Medium tertile	1.05 (0.57–1.92)	0.74 (0.37–1.46)
High tertile	1.00 (Ref)	1.00 (Ref)
**Meets Fruit & Vegetable Intake Recommendations**		
No	1.52 (0.52–4.46)	1.22 (0.39–3.81)
Yes	1.00 (Ref)	1.00 (Ref)
**Meets Dairy Intake Recommendations**		
No	0.93 (0.46–1.86)	0.69 (0.33–1.48)
Yes	1.00 (Ref)	1.00 (Ref)
**≥1 Sugar-Sweetened Beverage per Day**		
Yes	1.78 (0.91–3.49)	1.38 (0.69–2.79)
No	1.00 (Ref)	1.00 (Ref)
**Skips Breakfast**		
Yes	**3.40 (1.25–9.22)**	**3.73 (1.51–9.25)**
No	1.00 (Ref)	1.00 (Ref)
**Physical Activity Questionnaire for Children**		
Low tertile	2.16 (0.98–4.73)	1.86 (0.77–4.46)
Medium tertile	1.48 (0.56–3.90)	1.29 (0.46–3.63)
High tertile	1.00 (Ref)	1.00 (Ref)
**Physically Active without Instructor <4×/Week**		
Yes	2.22 (0.65–7.52)	1.84 (0.55–6.20)
No	1.00 (Ref)	1.00 (Ref)
**Physically Active with Instructor <4×/Week**		
Yes	1.64 (0.79–3.40)	1.56 (0.69–3.53)
No	1.00 (Ref)	1.00 (Ref)
**>2 h of Screentime**		
Yes	**1.83 (1.09–3.08)**	1.45 (0.83–2.52)
No	1.00 (Ref)	1.00 (Ref)
**Physically Active at Morning Recess**		
No	**2.32 (1.44–3.71)**	**2.05 (1.21–3.48)**
Yes	1.00 (Ref)	1.00 (Ref)
**Physically Active after School**		
No	**1.96 (1.16–3.31)**	**1.93 (1.07–3.48)**
Yes	1.00 (Ref)	1.00 (Ref)
**Combined Associations**		
**Diet Quality Index**		
Low tertile	1.66 (0.47–5.90)	1.47 (0.44–4.88)
Medium tertile	1.13 (0.32–3.95)	0.95 (0.28–3.17)
High tertile	1.00 (Ref)	1.00 (Ref)
**Physical Activity Questionnaire for Children**		
Low tertile	**2.31 (1.10–4.85)**	2.02 (0.85–4.80)
Medium tertile	1.63 (0.69–3.87)	1.42 (0.52–3.87)
High tertile	1.00 (Ref)	1.00 (Ref)
**>2 h of Screentime**		
Yes	0.59 (0.34–1.02)	0.74 (0.40–1.38)
No	1.00 (Ref)	1.00 (Ref)

## 4. Discussion

This research contributes to our understanding of the relationship between PA, HE and academic performance. We observed statistically significant positive associations for the main exposure of low diet quality with ELA academic performance and but not for PA with either academic outcome. We also found significant associations for some of the secondary exposure measures with academic performance in ELA or Mathematics, including PA after school and breakfast skipping. The individual effects of diet quality and PA on academic performance appeared to be independent from each other and there was no additive interaction between the two exposures.

The majority of academic literature has explored either the associations between diet and academic performance or PA and academic performance. Few studies have studied the associations of both health behaviours [24,25,26]. Our study allowed a comparison of the magnitude of relationships of diet and PA, respectively, with academic performance. Studying health behaviours simultaneously is important since many school-based health promotion interventions target several of these modifiable behaviours to improve health in the short term and reduce risk for cancer and chronic disease later in life [1,39]. A recent review suggested that patterns of diet and PA among children and youth cluster together in complex ways that are not well understood [40], suggesting that both health behaviours need to be assessed for their effect on academic performance independently. The few existing studies that examined both health behaviours reported positive associations with academic performance [24,25,26] but further research is needed to clarify the relationships. To the best of our knowledge, our study is the first to examine a potential additive interaction between diet quality and PA in their effect on academic performance, but there appeared to be none. The reversal of the effect of screentime on academic performance in Mathematics in the combined model with diet and PA was unexpected. We can only speculate that excess screentime may be a heterogeneous exposure with potential benefits (e.g., studying on the computer, programming) for academic performance in Mathematics in some children.

Our findings point to a potentially stronger association of diet with academic performance compared to PA but given the wide confidence intervals for the effect estimates this observation needs to be interpreted with caution. Many studies reporting positive associations with PA and academic performance used physical fitness or objectively measured PA (through pedometers or accelerometers) as opposed to self-reported measures [11,12,13,22]. It is possible that the stronger relationships for diet and academic performance compared to PA may due to a lower sensitivity of the PA questionnaire used in the current study. Another interesting finding is the observation that diet exposures appeared to show a stronger association with ELA than Mathematics academic performance, while PA exposures tended to have stronger associations with Mathematics. This finding is in keeping with results from a meta-analysis by Fedawa and Ahn (2011), which found that the largest effect of PA on school performance was on children’s mathematics achievements, followed by IQ and reading [11]. These observations may be sex-specific but the sample size of the current study did not permit a stratification by sex.

Our findings also highlight several potential points of intervention that may help to support learning goals of schools [9,11,12,13,41]. One important significant finding is the negative association between breakfast skipping and Mathematics performance. Canadian schools have historically been involved in supporting the nutritional needs of children through universal breakfast programs [42,43]. Previous reviews have found that the benefits of breakfast consumption were observed most on measures such as memory, errors on attention tasks and on-task behaviours [9,44] A systematic review of micronutrient supplementation assessed through randomized controlled trials suggested that supplementation may be associated with a marginal increase in fluid intelligence (based on reasoning abilities, comparable to Mathematics) but not with crystallized intelligence (verbal comprehension and vocabulary, comparable to ELA) [45]. Therefore, our study findings support the existing literature to suggest that breakfast programs may potentially help to reduce food insufficiency, improve nutritional status and support academic performance in Mathematics [7].

Research has also reported that different school-based PA strategies (e.g., physical education, extra-curricular activities, interscholastic sports and recess) may help support cognitive outcomes [11,12]. While more research is needed to understand the dose-response of PA and school performance, higher levels of PA appear to produce higher achievement outcomes [11] although there is some evidence to suggest that too much PA may result in poorer academic achievement, potentially as a result of children spending less time focusing on academic pursuits [16,17,18,19]. Our findings suggest that offering afterschool programs may help to support academic performance, as we observed that increased participation in PA after school was significantly associated with better academic performance in Mathematics. Building on previous literature, it is possible that afterschool programs could support the personal and social skills of children and provide an added opportunity for PA outside of school hours that leads to improved performance in school [11,46].

The current study has several strengths, including the use of a population-based sample of grade 4 to 6 students in one school board, and the use of academic performance scores obtained directly from the school board at three different time points over the academic year. Unfortunately, the small sample size reduced the power of our study to detect significant associations in our study. The response rate of 46% may have led to selection bias due to non-response. The use of non-response weights may have reduced but not completely eliminated non-response bias. It also appeared that competing priorities in schools also may have resulted in non-compliance of teachers in reminding students to return their consent forms. Also, considering the high obesity prevalence observed, the expected non-response from lower SES groups did not occur, suggesting a potentially lower effect of selection bias from this group. Measures to assess HE and PA were mostly based on validated instruments but self-reported response is inherently subjective and may be prone to error or bias. A further limitation is the cross-sectional nature of our data. Although measures were mostly based on validated instruments, self-reported response is inherently subjective and may be prone to error or bias [47,48]. Further, while we used a previously validated measure of PA through the PAQ-C, the scores do not allow for comparison with guidelines for PA (e.g., 30–60 min of moderate to vigorous PA).

## 5. Conclusions

There is increasing global advocacy for an emphasis on HE and PA in schools and there is an increasing evidence-base that describes their association with academic performance. However, initiatives to promote health often require time from school staff that is beyond their designated responsibilities [49]. With a growing workload, these activities become difficult for school staff to maintain. This is especially true when there is a lack of understanding of the importance and significance of healthy lifestyle behaviours to student learning, which in turn may limit the integration of health into the priorities of the education system [50,51]. Of more concern, and inconsistent with this substantive evidence-base, there has been an increasing trend to “cut-back” on activities in school that promote health and to view these activities as “add-ons” rather than activities that are central to the academic mission of schools. The results of this study adds to our growing understanding of the associations between health behaviours and academic performance and provides further rationale of the importance of health promotion interventions in supporting health and learning goals at schools. The findings are particularly policy relevant to education decision makers in Nova Scotia, where provincial academic scores are consistently lower than in the rest of the country. Further research should explore school-level differences to shed light on strategies to support improvements in both health and academic outcomes. Overall, our research demonstrates stronger support for the association between diet and academic performance compared to PA and suggests an emphasis on breakfast, and afterschool programs may help to support student health and learning.
